# Short-term effects of chemical and noise pollution during heat and cold waves on emergency hospital admissions in Madrid

**DOI:** 10.1007/s00484-025-02963-y

**Published:** 2025-06-30

**Authors:** R Ruiz-Páez, JA López-Bueno, J Díaz, MA Navas, C. Linares

**Affiliations:** 1 University of Alcalá, Madrid, Spain; 2 Present Address: Climate Change, Health and Urban Environment Reference Unit, Carlos III Institute of Health (Instituto de Salud Carlos III/ISCIII), Madrid, Spain

**Keywords:** Air pollution, Heat waves, Cold waves, Noise

## Abstract

Cold and heatwaves and air pollution have a joint, direct impact on population health. The aim of this study was to evaluate the combined impact of chemical air and noise pollution on hospital admissions during warm and cold months, with a breakdown by age group. We conducted a time-series study of unscheduled emergency admissions by specific causes in Madrid across the period 2013–2018. The independent variables were daily mean concentrations of PM_2.5_, PM_10_, NO_2_, tropospheric ozone, noise, and maximum and minimum temperatures. Relative and attributable risks were calculated using generalised linear models with a Poisson link. During the warm months, stronger associations were found between: ozone and admissions due to respiratory, circulatory and neurological causes; NO_2_ and admissions due to respiratory diseases; and PM and admissions due to dementia and Alzheimer’s disease. Heat had a greater effect on admissions due to neurological diseases. During the cold months, no association was found with any chemical air pollutant, except for NO_2_ and asthma-related admissions. By age, the greatest impacts were registered for PM, noise, cold and heat at ages ≥ 65 years, and for noise, NO_2_ and cold at ages ≤ 14 years.

**Supplementary Information:**

The online version contains supplementary material available at 10.1007/s00484-025-02963-y.

## Introduction

Climate change has led to an increase in the frequency and duration of extreme temperature events, with direct impacts on population health. By extension, air pollution, which is closely linked to climate change, also has a significant influence on health (Fuller et al. 2022; IPCC 2023; European Environment Agency 2023). At the most recent United Nations Climate Change Conference (COP 28), emphasis was laid on the imperative need to maintain the increase in the global temperature below 1.5 °C, as laid down in the Paris Accord (UNFCCC 2023). Even so, the year 2023 ranked as the hottest on record to date (WMO 2023). Many studies have examined the impacts of cold and heatwaves on health (Ebi et al. 2021; Lo et al. 2024; Weilnhammer et al. 2021), with it being estimated that increases of 1 °C are correlated with an 18% increase in heat-related disease morbidity (Faurie et al. 2022).

With the aim of mitigating the impacts of extreme temperatures on health, a heatwave surveillance and control plan, as well as another similar plan for the effects of cold on health have been implemented in Madrid (Dirección General de Salud Pública 2023a, b). Yet, both plans are exclusively activated by reference to ambient temperatures, without the effects of air pollution being taken into consideration or guidelines being adjusted in line with the periods of greatest pollution.

Given that air pollution is considered to be the principal environmental risk factor for health, it is essential that it be incorporated into public health prevention plans (WHO 2022). Although many countries have introduced regulations governing air pollution levels, according to the latest European Environmental Agency report, in 2021 air pollutant concentrations in Europe continued to exceed the World Health Organisation (WHO) guideline values (European Environment Agency 2023). In addition to chemical pollution, we are also faced with noise pollution, exposure to which increases the risk of cardiovascular, metabolic, mental and reproductive diseases (Chen et al. 2023; Zaman et al. 2022; Sivakumaran et al. 2022; Hegewald et al. 2020). Indeed, a previous study in Madrid attributed more than 5,600 annual natural-cause hospital admissions to diurnal noise (Ruiz-Páez et al. 2023a).

While earlier research analysed the interaction between extreme temperature events and air pollution (Park et al. 2023; Stafoggia et al. 2023; Ni et al. 2023; Grigorieva and Lukyanets 2021), recent studies on the synergic effects are very varied and tend to focus on specific pollutants and diseases. Studies that have examined the effects of fine particulate matter having a diameter of 10 microns or less (PM_10_) during high temperature episodes have reported an increased risk associated with respiratory (Areal et al. 2022) and cardiovascular diseases (Parry et al. 2019). Another study which sought to ascertain how temperature modifies PM and NO_2_ concentrations, described an increase in their impact on respiratory diseases during low temperature episodes (Shen et al. 2023). With respect to ozone, its atmospheric concentration tends to increase in times of high temperatures, inasmuch as its formation depends on the presence of sunlight (Wang et al. 2023). It is therefore plausible to expect that its impact would be more pronounced during heatwave episodes. In the case of cold waves, a recent study described the increased risk posed by PM_2.5_ concentrations in childhood asthma during low-temperature events (Landguth et al. 2023).

It is important to highlight the fact that extreme temperature conditions and chemical air and noise pollution exert significant effects on the more vulnerable population segments, such as children and the elderly (van Daalen et al. 2022; European Environment Agency 2023).

The aim of this study was thus to analyse the combined effect of chemical air and noise pollution on unscheduled hospital admissions during cold and heat waves, with a breakdown by age group. The findings obtained could well contribute to the drawing up of prevention strategies that guarantee more effective protection of health.

## Materials and methods

### Study design

Similar to previous studies (Ruiz-Páez et al. 2023a), a retrospective, longitudinal and ecological time series study was performed in the present study. The relationship between unscheduled emergency hospital admissions in the Madrid Region (Comunidad de Madrid) and the presence of chemical air and noise pollution was analyzed for both summer and winter months and for different age groups.

### Dependent variables

The dependent variables were the number of daily emergency hospital admissions at all hospitals in the Madrid Region across the period 1 January 2013 to 31 December 2018. The International Classification of Diseases, Ninth Edition (ICD-9) and Tenth Edition (ICD-10) codes used were as follows: respiratory causes (ICD-9: 460–519 and ICD-10: J00-J99); acute upper respiratory tract infection (ICD-9: 034.0, 460–465 and ICD-10: J00-J06); asthma (ICD-9: 493 and ICD-10: J45); pneumonia (ICD- 9: 481–483 and ICD-10: J13-J16, J18.1, A48.1); circulatory causes (ICD-9: 390–459 and ICD-10: I00-I99); acute cerebrovascular accident (ICD-9: 431–434 and ICD-10: I61-I64); acute myocardial infarction (ICD-9: 410 and ICD-10: I21-I22); ischaemic heart disease (ICD-9: 414.8, 414.9, 429.9 and ICD-10: I51.9, I25.9); Parkinson’s disease (ICD-9: 332.0 and ICD-10: G20); dementia (ICD-9: 290, 294.2 and ICD-10: F02, F03); Alzheimer’s disease (ICD-9: 331.0 and ICD-10: G30); multiple sclerosis (ICD-9: 340 and ICD-10: G35). The data were sourced from the Ministry of Health’s Minimum Basic Data Set (Conjunto Mínimo Básico de Datos/CMBD) under a confidential microdata assignment agreement from the National Statistics Institute (Instituto Nacional de Estadística/INE). We broke down the data by age group, i.e., over 65 years and under 14 years, and in the case of minors, limited the analysis to respiratory-cause admissions, in view of the fact that incidence of admissions due to circulatory and neurological diseases in this age group is practically non-existent.

### Independent variables

Chemical air pollutants: average daily concentrations (μg/m^3^) of fine particulate matter having a diameter of 2.5 μm or less (PM_2.5_), fine particulate matter having a diameter of 10 μm or less (PM_10_), nitrogen dioxide (NO_2_), and tropospheric ozone (O_3_). These data were supplied by the Ministry for Ecological Transition and Demographic Challenge (Ministerio para la Transición Ecológica y Reto Demográfico) across the 6-year study period.

Noise pollution: average daily noise levels (dB(A)) of diurnal noise measured from 7am to 11pm (L_Aeq,7-23h_), nocturnal noise from 11pm to 7am (L_Aeq,23-7h_), and noise throughout the day (L_Aeq,24h_). Noise level data were obtained from the Madrid Municipal Permanent Acoustic Pollution Monitoring Grid (Red Fija de Control de la Contaminación Acústica del Ayuntamiento de Madrid) and the AENA Airports Acoustic Pollution Monitoring Grid (Red de Control de la Contaminación Acústica de AENA) for the period 1 January 2014 to 31 December 2018 (5 years).

Meteorological control variables: daily meteorological variables of maximum and minimum temperatures (°C), number of hours of sunlight, wind (km/h), air pressure (hPa) and relative humidity (%). These data were sourced from the Madrid-Retiro Observatory (reference observatory for the Madrid Region) and the State Meteorology Agency (Agencia Estatal de Meteorología/AEMET).

Appendix 1 shows the geographical location of this meteorological observatory and other stations included in the study, such as those monitoring noise and air pollution.

Other control variables: autoregressive nature of the dependent variables, trend of the series, national and regional Public Holidays, and seasonality of the series (annual, six-monthly, four-monthly and quarterly).

Seasons: we worked with data for the six years, grouped into two periods, namely, summer months from 1 May to 30 September (N= 918 days) and winter months from 1 November to 31 March (N= 907 days). These dates were chosen in order to cover the periods of warm and cool temperatures in the Madrid Region, and thereby enlarge the sample size (AEMET 2023).

### Statistical analyses

The functional relationship between the environmental variables analysed and unscheduled emergency admissions shows a linear distribution without threshold (Linares et al. 2018), except for temperature and ozone which display a quadratic distribution (Díaz et al. 1999; Alberdi et al. 1998) and were thus both parameterised.

To identify cold wave and heatwave days, we used the temperatures defined by the Ministry of Health, with 34 °C being taken as the heatwave threshold (Ministerio de Sanidad 2023a) and 1.9 °C as the cold-wave threshold (Ministerio de Sanidad 2023b). Two new variables were created:

$$\:{T}_{heat}=0,if{T}_{max}<34$$

$$\:T_{heat}=T_{max}-34{}^\circ C,ifT_{max}>34$$

$$\:{T}_{cold}=0,if{T}_{min}>1.9$$

$$\:T_{cold}=1.9{}^\circ C-T_{min},ifT_{min}<1.9$$

In the case of ozone, we calculated the minimum value of the cubic function by means of its corresponding fit to a 3nd-order polynomial (107.5 µg/m^3^). To identify the days on which ozone exceeded this value, a new variable was created:

$$\:{O}_{3}high=0,if\left[{O}_{3}\right]<107.5{\upmu\:}g/{m}^{3}$$

$$\:{O}_{3}high={O}_{3}-107.5{\upmu\:}g/{m}^{3},if\left[{O}_{3}\right]>107.5{\upmu\:}g/{m}^{3}$$

With regard to noise, the fact that there are no situations of total absence of noise led us to define the WHO guideline values as thresholds, corresponding to 50 dB(A) for diurnal noise, 45 dB(A) for nocturnal noise, and 53 dB(A) for noise throughout the day (Clark and Paunovic 2018; Gómez González et al. 2023).

A number of studies have shown that the short-term effect of pollution and meteorological conditions on morbimortality may not become apparent until days afterwards (Ortiz et al. 2017; Linares et al. 2018, 2025; Díaz et al. 2002, 2018; González et al. 2001; Ruiz-Páez et al. 2025). We therefore created lags for the following variables: in the case of PM_2.5_, PM_10_, NO_2_ and noise, lags of 5 days; in the case of ozone, lags of 8 days; in the case of heat, lags of 5 days; and in the case of a difference in air pressure, lags of 8 days. Since there is no current literature on the lagged effect of cold or humidity analysed jointly with the other meteorological variables, we used lags of 14 days as in previous studies (Ruiz-Páez et al. 2023a; Gómez González et al. 2023; Calle-Martínez et al. 2023; Egea et al. 2023).

To analyse the impact of chemical and noise pollution on emergency hospital admissions, we fitted generalised linear models (GLMs) with a Poisson regression link, and controlled for overdispersion. The backward method was applied to obtain the final model, using only those variables that had a statistically significant level of *p* < 0.05. The relative risks (RRs) of hospital admissions were calculated for every increase of 10 µg/m^3^ in the case of PM_2.5_, PM_10_, NO_2_ and ozone, and increase of one-unit in the case of heat and cold. Lastly, the attributable risks (ARs) of hospital admissions due to chemical and noise pollution were calculated using the equation: AR = (RR– 1)/RR * 100 (Coste and Spira 1991).

All the statistical analyses were performed using the STATA/MP v18.0 and Microsoft Excel 2021 computer software package.

## Results

Table 1 shows the descriptive analysis of the variables related with chemical air and noise pollution across the year, and specifically during the summer and winter months. The variables of noise and PM_2.5_ exhibited a notable degree of stability throughout the year. Ozone, in contrast, displayed a marked seasonal behaviour, with an increase in its values during the hot sunny months. During the warm months, an increase was likewise observed in PM_10_ levels. With respect to the meteorological variables, these reflected the typical seasonality of the hot, dry summers and mild, rainy winters that characterise the region (AEMET 2023). Table 2 shows the distribution of mean daily hospital admissions, classified by respiratory, circulatory and neurological causes (Parkinson’s disease, dementia, Alzheimer’s disease and multiple sclerosis), with a breakdown by summer/winter months and age group. In general terms, respiratory-cause admissions were predominant in the complete series. On analysing the differences in the averages of daily admissions by season with respect to the complete series, a decrease was observed during the hot season and an increase during the cold season. This variation was especially pronounced in respiratory-cause admissions. No important variations were observed in neurological-cause admissions by season of the year. In terms of age groups, in the over-65 group respiratory-cause admissions were observed to register a mean number of daily admissions very similar to that for circulatory causes.

**Table 1:** Description of air and acoustic pollution and the meteorological conditions for the 2013–2018 series, Madrid

	Whole serie (*N* = 2191)		Warm months^b^ (*N* = 918)		Cold months^c^ (*N* = 907)	
	X ± SD	Min/Max	X ± SD	Min/Max	X ± SD	Min/Max
Air pollution:						
PM_2,5_ (µg/m^3^)	10.3 ± 4.7	3.2/33.1	10.3 ± 3.8	3.4/33.1	10.7 ± 5.8	3.2/31.5
PM_10_ (µg/m^3^)	19 ± 9.7	2.9/85.7	20.9 ± 9.2	6.5/85.7	17.3 ± 9.9	2.9/85.1
NO_2_ (µg/m^3^)	30.7 ± 14.5	5.8/90.9	24.1 ± 8.8	7.7/59.1	37.6 ± 16.5	5.8/90.9
O_3_ (µg/m^3^)	56.4 ± 23.0	6.1/113.7	74.6 ± 14.2	30.6/113.7	39.4 ± 18.0	6.1/92.1
Acoustic pollution:						
Diurnal noise (dB(A))^a^	56.2 ± 2.3	48.1/62.0	56.0 ± 2.1	49.3/61.7	56.6 ± 2.2	48.6/62.0
Nocturnal noise (dB(A))^a^	49.7 ± 2.1	41.5/58.1	49.7 ± 1.9	44.4/56.6	50.1 ± 2.2	42.7/58.1
24 h noise (dB(A))^a^	54.9 ± 2.2	46.8/61.0	54.6 ± 2.1	46.8/60.7	55.4 ± 2.1	47.6/61.0
Meteorological conditions:						
Relative humidity (%)	59.7 ± 16.3	19.0/95.2	47.9 ± 10.9	19.0/93.9	70.5 ± 13.1	31.5/95.2
Atmospheric pressure (hPa)	940.7 ± 6.0	911.8/962.6	939.8 ± 3.3	923.9/950.6	941.9 ± 7.9	911.8/962.6
T _max_ (°C)	21.1 ± 9.1	2.8/40.0	29.7 ± 5.5	9.2/40.0	12.6 ± 3.7	2.8/26.7
T _min_ (°C)	11.1 ± 6.8	−3/25.9	17.2 ± 4.3	4.5/25.9	4.7 ± 3.0	−3/14.1
Wind (km/h)	6.4 ± 3.0	0.0/18.7	6.7 ± 2.0	1.8/13.5	6.2 ± 3.9	0.0/18.7
No. sunlight hours per day	8.1 ± 4.3	0.0/14.4	10.8 ± 3.3	0.0/14.4	5.7 ± 3.6	0.0/12.2

*X* media, *SD* standard deviation. ^a^ Period 2014–2018. ^b^ Warm months: from May 1 to September 30. ^c^ Cold months: from November 1 to March 31

**Table 2:** Description of daily emergency hospital admissions for the 2013–2018 series, Madrid

	Whole serie		Seasons				Age groups			
			Warm months		Cold months		≥ 65 years		≤ 14 years	
	X ± SD	Min/Max	X ± SD	Min/Max	X ± SD	Min/Max	X ± SD	Min/Max	X ± SD	Min/Max
All respiratory-causes	185.7 ± 77.4	48/554	127.5 ± 40.6	48/239	249.7 ± 69	120/554	115.5 ± 51.3	30/381	33.8 ± 23	0/134
URI	5.5 ± 2.7	0/16	4.9 ± 2.7	0/16	6.1 ± 2.6	0/16	0.7 ± 0.9	0/6	3.2 ± 2.1	0/12
Asthma	10 ± 5.3	0/30	7.2 ± 5.1	0/29	12.5 ± 4.7	2/30	3.8 ± 2.9	0/19	3 ± 2.5	0/15
Pneumonia	6 ± 3.4	0/26	4.3 ± 2.5	0/16	7.8 ± 3.6	0/26	4 ± 2.6	0/21	0.4 ± 0.7	0/4
All circulatory-causes	149 ± 33.7	54/230	135 ± 30.6	54/207	160.2 ± 32.5	82/230	115.4 ± 26.9	36/181		
Ischemic heart disease	0.3 ± 0.6	0/4	0.4 ± 0.6	0/4	0.3 ± 0.6	0/4	0.3 ± 0.5	0/3		
MI	14 ± 4.2	1/31	12.5 ± 3.9	1/28	15.2 ± 4	4/31	8.1 ± 3.2	0/20		
CVA	23.2 ± 5.7	4/43	21.7 ± 5.7	7/43	24.3 ± 5.4	4/43	18.1 ± 4.9	4/37		
Parkinson	0.3 ± 0.5	0/4	0.3 ± 0.5	0/3	0.3 ± 0.6	0/4	0.2 ± 0.5	0/4		
Dementia	0.5 ± 0.7	0/4	0.5 ± 0.7	0/4	0.5 ± 0.7	0/4	0.5 ± 0.7	0/4		
Alzheimer	0.5 ± 0.7	0/4	0.5 ± 0.8	0/4	0.5 ± 0.7	0/4	0.5 ± 0.7	0/4		
Multiple sclerosis	0.5 ± 0.7	0/5	0.5 ± 0.7	0/4	0.4 ± 0.7	0/4	0 ± 0.2	0/1		

*URI* Acute upper respiratory infections, *MI* Acute myocardial infarction, *CVA* cerebral vascular accident, *DS* desviación estándar, *X* Media, *SD* standard deviation. ^a^ Warm months: from May 1 to September 30. ^b^ Cold months: from November 1 to March 31

On analysing the RRs and ARs of the statistically significant variables yielded by the Poisson regression models for hospital admissions (Tables 3, 4 and 5), the greatest effect was observed for ozone concentrations in the summer months, among both the over-65 and under-14 age groups. During the winter months, a number of variables of statistical importance were identified but were associated with lower relative and attributable risks, with the exception of the impact associated with cold in admissions due to Parkinson’s disease and ischaemic heart disease. Appendix 2 shows the RRs and ARs in respect of data for the whole series, without any breakdown by season.

**Table 3:** Effect of environmental variables on emergency hospital admissions according to warm and cold months

	Warm months		Cold months		
	RR (95% CI)	AR (95% CI)		RR (95% CI)	AR (95% CI)
All respiratory-causes					
NO_2_ (3)	1.01 (1.00–1.02)	0.94 (0.10–1.77)	Leqd (0)	1.00 (1.00–1.01)	0.42 (0.19–0.65)
O_3_ (1)	1.25 (1.04–1.51)	20.13 (3.96–33.57)	T.cold (3)	1.02 (1.01–1.03)	1.88 (1.25–2.50)
T.heat (2)	1.01 (1.00–1.03)	1.27 (0.06–2.46)	T.cold (5)	1.02 (1.01–1.02)	1.62 (1.01–2.22)
			T.cold (12)	1.02 (1.01–1.02)	1.72 (1.13–2.32)
URI					
NO_2_ (3)	1.05 (1.02–1.09)	5.19 (1.61–8.63)	Leqn (4)	1.01 (1.00–1.03)	1.41 (0.07–2.72)
Asthma					
O_3_ (7)	3.15 (1.36–7.31)	68.29 (26.55–86.31)	NO_2_ (4)	1.02 (1.01–1.04)	2.34 (1.04–3.62)
			T.cold (5)	1.03 (1.00–1.05)	2.67 (0.26–5.02)
Pneumonia					
NO_2_ (5)	1.05 (1.01–1.09)	4.39 (0.53–8.10)	Leqn (1)	1.02 (1.00–1.03)	1.59 (0.36–2.80)
			Leqn (5)	1.01 (1.00–1.03)	1.42 (0.22–2.61)
			T.cold (5)	1.06 (1.02–1.09)	5.35 (2.20–8.39)
All circulatory-causes					
O_3_ (2)	1.20 (1.01–1.43)	16.59 (0.61–30.00)	Leqd (0)	1.00 (1.00–1.01)	0.41 (0.13–0.69)
O_3_ (8)	1.25 (1.05–1.49)	19.92 (4.55–32.81)	T.cold (4)	1.01 (1.00–1.02)	1.24 (0.45–2.01)
Ischemic heart disease					
Leq24 (3)	1.08 (1.01–1.15)	7.06 (0.97–12.77)	T.cold (5)	1.15 (1.01–1.30)	12.91 (1.12–23.30)
MI					
O_3_ (1)	1.81 (1.06–3.09)	44.71 (5.67–67.59)	Leq24 (0)	1.01 (1.00–1.02)	0.96 (0.05–1.86)
O_3_ (8)	1.93 (1.13–3.29)	48.20 (11.84–69.57)	T.cold (7)	1.03 (1.00–1.05)	2.68 (0.23–5.06)
Leqd (0)	1.01 (1.00–1.03)	1.43 (0.35–2.49)			
CVA					
O_3_ (1)	1.80 (1.20–2.69)	44.37 (16.83–62.80)	Leqd (0)	1.01 (1.00–1.02)	1.01 (0.30–1.72)
Leq24 (3)	1.01 (1.00–1.02)	0.89 (0.08–1.70)			
Parkinson					
Leqd (1)	1.08 (1.02–1.16)	7.77 (1.72–13.45)	Leqd (3)	1.08 (1.01–1.16)	7.74 (1.35–13.72)
T.heat (3)	1.29 (1.09–1.54)	22.73 (8.26–34.92)	T.cold (8)	1.21 (1.04–1.42)	17.66 (3.74–29.56)
Dementia					
PM_2.5_ (3)	1.31 (1.03–1.68)	23.76 (2.59–40.33)	Leqd (0)	1.07 (1.01–1.12)	6.16 (1.40–10.70)
Leqn (1)	1.08 (1.03–1.15)	7.79 (2.50–12.79)			
Alzheimer					
PM_10_ (0)	1.12 (1.03–1.23)	10.84 (2.57–18.41)			
T.heat (3)	1.17 (1.03–1.34)	14.72 (2.66–25.29)			
Multiple sclerosis					
O_3_ (2)	6.85 (1.03–45.66)	85.41 (2.74–97.81)	T.cold (6)	1.13 (1.00–1.26)	11.15 (0.38–20.76)
Leqd (5)	1.05 (1.00–1.11)	5.15 (0.26–9.80)			

Relative risks (RRs) and attributable risks (ARs) with their respective 95% Cls of the significiant independent variables. Increases for every 10 µg/m3, above the 8-h ozone threshold of 107.5 µg/m3. Lags shown in brackets. *URI* Acute upper respiratory infections, *MI* Acute myocardial infarction, *CVA* cerebral vascular accident, *Leqd* LAeq,7–23 h, *Leqn* LAeq,23–7 h, *Leq24* LAeq,24 h

**Table 4:** Effect of environmental variables on respiratory admissions by age group

	≥ 65 years		≤ 14 years		
	RR (95% CI)	AR (95% CI)		RR (95% CI)	AR (95% CI)
All respiratory-causes					
Leqd (0)	1.00 (1.00–1.00)	0.24 (0.02–0.46)	NO_2_ (3)	1.01 (1.00–1.02)	0.83 (0.18–1.48)
T.heat (2)	1.04 (1.02–1.05)	3.66 (2.32–4.97)	O_3_ (5)	2.94 (1.65–5.25)	66.00 (39.28–80.96)
T.cold (3)	1.03 (1.02–1.04)	2.88 (2.14–3.62)	O_3_ (8)	2.17 (1.13–4.16)	53.86 (11.38–75.98)
T.cold (5)	1.02 (1.01–1.02)	1.66 (0.92–2.40)	Leqd (3)	1.01 (1.00–1.01)	0.66 (0.28–1.04)
T.cold (12)	1.03 (1.02–1.04)	2.84 (2.14–3.53)	T.cold (8)	1.02 (1.01–1.04)	2.27 (0.75–3.76)
URI					
O_3_ (8)	14.62 (2.40–89.21)	93.16 (58.25–98.88)	NO_2_ (4)	1.03 (1.01–1.06)	3.36 (1.28–5.40)
Leqn (3)	1.03 (1.01–1.06)	3.33 (0.71–5.87)	Leqn (4)	1.02 (1.01–1.03)	1.80 (0.50–3.07)
T.cold (3)	1.18 (1.00–1.38)	14.97 (0.09–27.64)			
T.heat (4)	1.10 (1.01–1.20)	9.29 (0.91–16.96)			
Asthma					
Leqn (3)	1.01 (1.00–1.03)	1.44 (0.28–2.58)	NO_2_ (4)	1.03 (1.01–1.05)	2.78 (0.51–4.99)
T.cold (5)	1.05 (1.01–1.09)	4.81 (1.10–8.39)	Leqd (3)	1.02 (1.01–1.03)	1.79 (0.52–3.04)
Pneumonia					
T.heat (3)	1.08 (1.01–1.15)	7.21 (0.61–13.37)			
T.cold (5)	1.05 (1.01–1.09)	4.90 (1.42–8.26)			
T.cold (11)	1.07 (1.03–1.10)	6.11 (2.75–9.36)			

**Table 5:** Effect of environmental variables on circulatory and neurological admissions by age group

	≥ 65 years	
	RR (95% CI)	AR (95% CI)
All circulatory-causes		
O_3_ (1)	1.35 (1.11–1.64)	25.70 (9.61–38.92)
O_3_ (4)	1.48 (1.19–1.84)	32.36 (15.99–45.55)
Leqd (0)	1.00 (1.00–1.01)	0.35 (0.13–0.57)
T.cold (4)	1.01 (1.00–1.02)	1.15 (0.19–2.10)
T.cold (6)	1.01 (1.00–1.02)	1.25 (0.32–2.17)
Ischemic heart disease		
Leq24 (3)	1.07 (1.03–1.13)	6.95 (2.59–11.12)
MI		
O_3_ (1)	2.26 (1.16–4.42)	55.79 (13.69–77.35)
Leqd (0)	1.01 (1.00–1.02)	0.93 (0.13–1.72)
T.cold (7)	1.05 (1.01–1.08)	4.33 (1.13–7.43)
CVA		
O_3_ (1)	1.98 (1.27–3.09)	49.46 (21.02–67.66)
Leqd (0)	1.01 (1.00–1.01)	0.90 (0.36–1.44)
Parkinson		
Leqd (3)	1.06 (1.01–1.11)	5.52 (1.21–9.65)
T.heat (3)	1.23 (1.02–1.48)	18.61 (1.87–32.49)
Dementia		
PM_2.5_ (3)	1.16 (1.02–1.31)	13.49 (2.11–23.54)
Alzheimer		
PM_10_ (0)	1.07 (1.01–1.14)	6.39 (0.51–11.93)
T.heat (3)	1.20 (1.04–1.37)	16.34 (4.30–26.86)

Relative risks (RRs) and attributable risks (ARs) with their respective 95% Cls of the significiant independent variables. Increases for every 10 µg/m3, above the 8-h ozone threshold of 107.5 µg/m3. Lags shown in brackets. *Leqd* LAeq,7–23 h, *Leqn* LAeq,23–7 h, *Leq24* LAeq,24 h

**Fig. 1:**
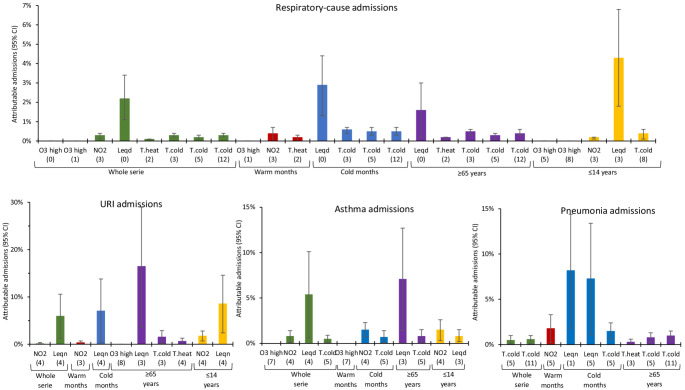
Percentage of attributable admissions with their respective 95% Cls for the significiant independent variables in respiratory-cause admissions. Increases for every 10 μg/m^3^ above the 8-h ozone threshold of 107.5 μg/m^3^. Lags shown in brackets. Leqd: L_Aeq,7-23h_, Leqn: L_Aeq,23-7h_, Leq24: L_Aeq,24h_. URTIs: Acute upper respiratory tract infections

**Fig. 2:**
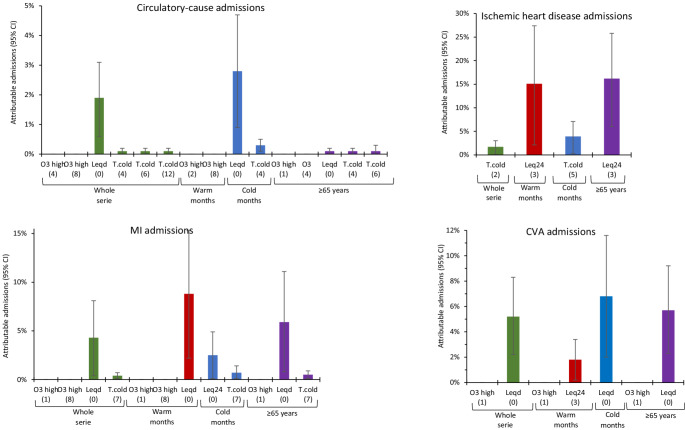
Percentage of attributable admissions with their respective 95% Cls for the significiant independent variables in circulatory-cause admissions. Increases for every 10 μg/m^3^ above the 8-h ozone threshold of 107.5 μg/m^3^. Lags shown in brackets. Leqd: L_Aeq,7-23h_, Leqn: L_Aeq,23-7h_, Leq24: L_Aeq,24h_. MI: Acute myocardial infarction. ACVA: Acute cerebrovascular accident

**Fig. 3:**
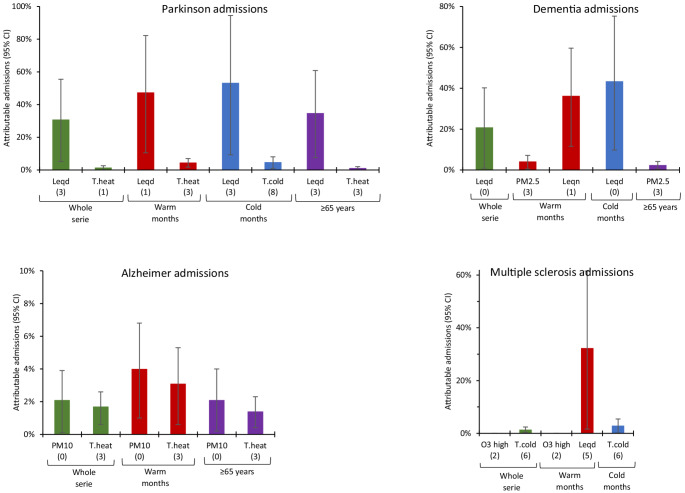
Percentage of attributable admissions with their respective 95% Cls for the significant independent variables in neurological-cause admissions. Increases for every 10 μg/m^3^ above the 8-h ozone threshold of 107.5 μg/m^3^. Lags shown in brackets. Leqd: L_Aeq,7-23h_, Leqn: L_Aeq,23-7h_, Leq24: L_Aeq,24h_

Figures 1, 2 and 3 show the percentage of admissions attributable to chemical and noise pollution. It is important to note here that, despite the fact that the highest RRs and ARs of all the variables corresponded to ozone concentrations, calculation of the proportion of admissions for which ozone was responsible showed that its impact was in fact minimal. Noise, in contrast, proved to be the pollutant with the highest proportion of attributable admissions.

During the summer months, 30–50% of admissions due to neurological causes (except for Alzheimer’s disease, for which there was no effect) and 2–15% of admissions due to circulatory causes were attributed to noise. In the case of admissions due to respiratory causes, acute upper respiratory tract infections (URTIs) and pneumonia, NO_2_ was the main air pollutant responsible, accounting for 2% of attributable admissions in pneumonia. PM concentrations influenced admissions due to dementia and Alzheimer’s disease, accounting for 4% of attributable admissions.

During the winter months, noise levels again ranked as the main pollutant responsible for admissions due to respiratory causes, circulatory causes and, in greater measure, for admissions due to neurological causes. 1.5% of asthma-related admissions were attributed to NO_2_ concentrations.

When the impact of pollutants on attributable admissions was analysed by age group, noise was the pollutant with the highest percentage of attributable admissions among the over-65 segment. With respect to admissions due to neurological causes in this age group, this effect was only evident in Parkinson’s disease, albeit to a noteworthy degree. PM concentrations were responsible for 2% of admissions due to dementia and Alzheimer’s disease.

Lastly, in the under-14 age group, a greater proportion of admissions in respiratory diseases and URTIs were once again attributed to noise levels (4% and 8% of attributable admissions respectively). NO_2_ accounted for 2% of admissions due to URTIs and asthma.

## Discussion

The results in Table 1 show that there is an increase in tropospheric ozone levels during the summer months. This increase, which is essentially in an urban atmosphere, is generated by photochemical reactions as well as PM_10_ concentrations. Although there is normally a higher PM_10_ concentration in the winter months, our study shows an increase during the summer which is attributable to the greater incidence of conditions of suspended dust particles in the atmosphere. A study published on the incidence of Saharan dust intrusion and forest fires in Spain during this same period (Ruiz-Páez et al. 2024) shows that in Madrid, the phenomenon with the highest incidence and potentially increasing PM_10_ concentrations in summer months is Saharan dust intrusion, which occurred on 741 days out of a total of 3,652, with PM_10_ concentrations increasing from average values ​​of 18.4 µg/m^3^ on days without intrusion to 32.7 µg/m^3^ on days with intrusion. PM_10_ intrusion from biomass combustion in Madrid during this same period occurred on only 320 days, and PM_10_ concentrations increased from 21.0 µg/m^3^ on days without PM10 advection due to biomass combustion to 24.4 µg/m^3^ on days with combustion.

In addition, NO_2_ concentrations tend to increase during the winter months, in line with the situation described in previous reports (MITECO 2023).

Heatwaves generate favourable conditions for the accumulation of chemical air pollutants (WMO 2023). Our study found that the following pollutants had noteworthy effects during the summer months: ozone concentrations overall; NO_2_ concentrations on respiratory admissions; and PM concentrations on neurological admissions. That said, however, admissions attributable to ozone were lower than 0.05%, probably due to the high threshold value used for fitting the ozone data, which was only exceeded on 0.3% of days in the series. Unlike the findings obtained in our study, a recent meta-analysis of respiratory-cause hospitalisations during the hot season registered an OR of 1.011 (95% CI: 0.999–1.040) for ozone, 1.015 (95% CI: 0.995–1.036) for PM10, although these estimations did not reached statically significance and inconsistent results for NO2 (Areal et al. 2022).

With respect to our finding of the impact of PM concentrations on neurological-cause admissions, a literature review of the effects of pollution on the central nervous system highlighted an increased risk of relapse of multiple sclerosis after exposure to ozone during the hot season, as well as an increase in the impact of NO_2_ and PM_10_ during the cold season (Alhussaini et al., 2023; Vojinović et al. 2015). It is important to note here that despite the amply documented health impacts of ozone, NO_2_ and PM, there is nonetheless a high degree of heterogeneity in the results when it comes to ascertaining whether high temperatures have an influence on such associations. This variability can be attributed to the diversity of the definitions of high temperatures used (Conti et al. 2022) and the varying degree of population adaptation to heat conditions (Navas-Martín et al. 2024). Moreover, since 2015, regulations have been in force in Madrid, which govern atmospheric levels of PM and have succeeded in maintaining concentrations below WHO guideline threshold values (Comunidad de Madrid 2024).

During the winter months, our study found no statistically significant association between any chemical air pollutant and hospital admissions, save for NO_2_ in the case of admissions due to asthma. The remaining chemical pollutants showed no statistical significance, in line with the results of two meta-analyses conducted into the effect of pollution and low temperatures on admissions due to respiratory (Areal et al. 2022) and cardiovascular diseases (Wu et al. 2022). A study undertaken in a cold city in China, with a mean temperature of 7˚C, reported that increases of 10 µg/m^3^ in PM_2.5_ and NO_2_ led to respective increases of 0.31% and 0.95% in respiratory admissions (Jia et al. 2022).

The impact of cold is intensified during the winter months, with 3–5% of admissions due to ischaemic heart disease, multiple sclerosis and Parkinson’s disease being attributed to cold. While an overview of reviews identified an association between cold and cardiovascular morbidity, it neither found evidence of effects related with respiratory diseases nor examined neurological disease (Song et al. 2017). In the case of heat, our study shows an increased risk of admission due to respiratory causes, and in particular, an increased risk of admission due to Parkinson’s and Alzheimer’s diseases during the summer months among the population over the age of 65 years. These findings are in line with previous studies conducted in Madrid (Culqui et al. 2017; Linares et al. 2016, 2017). It is important to stress that heat does not affect the population under the age of 14 years, as noted above (Díaz et al. 2015).

Given that air pollution particularly affects the vulnerable population, our study analysed the effect on people over the age of 65 years. Data on the elderly population drawn from two studies undertaken in Italy and China agree that PM is associated with higher risks of hospitalisation due to respiratory disease (Renzi et al. 2022) and to digestive, musculoskeletal and genitourinary diseases (Gu et al. 2020). These findings are similar to the results obtained in our study. It is important to underscore the importance of regulatory measures aimed at controlling air pollution levels. In the absence of designated limits, (and thus with considerably higher atmospheric levels) it is likely that admissions attributable to PM might be even higher.

There is increasingly more evidence on the adverse effects that air pollution has on children’s health. A narrative review focusing on the impacts of pollution reports associations with various paediatric diseases, including respiratory, allergic and neuroendocrine diseases, among others (Lin et al. 2023). In our study, analysis of the effect of pollution on respiratory diseases showed that 2% of admissions due to URTIs and asthma were attributable to NO_2_ concentrations. In contrast, no statistically significant association with PM concentrations was identified, despite the fact that a recent meta-analysis on the relationship between PM and URTIs found a significant association (Ziou et al. 2022). Another study conducted in China reported a significant association between PM, NO_2_ and admissions due to lower respiratory tract infections (Zhang et al. 2023). It is important to note the disparities in environmental risks in terms of hospital admission due to respiratory causes between people aged over 65 years and those aged under 14 years (Table 4): in the over-65 age group, cold, heat, noise and ozone (solely in the case of URTIs) were observed to increase the risk of hospital admission, possibly due to exacerbation of pre-existing diseases (Meade et al. 2020); in the under-14 age group, on the other hand, NO_2_ emerges as a pollutant with a significant relative risk. This association might be attributable, not only to exacerbation of respiratory diseases at paediatric age, but also to a direct irritant effect of pollutants on respiratory tracts, as well as greater exposure to pollution, given that children spend more time outdoors and are physically closer to the ground, where the pollutants are mostly concentrated (WHO 2018). It is crucial to stress that exposure to pollution during childhood can influence the development of respiratory disease at adult age (Mocelin et al. 2022). Hence, it is of the utmost importance to ensure that recommendations targeted at protecting the childhood population are incorporated into pollution prevention plans.

A notable finding of our study is the considerable percentage of admissions attributable to noise levels in both age groups, and in particular, the high percentage attributable to noise pollution in admissions due to dementia and Parkinson’s disease. In a systematic review of the influence of noise on health, the findings relating to dementia concluded that there was limited evidence of the impact of traffic noise (Clark et al. 2020). Yet one should nevertheless highlight the fact that the synergic effect with extreme temperatures was not explored. Previous studies undertaken in the Madrid Region have analysed the impact of air and noise pollution on hospital admissions, without differentiating between cold and heatwaves (Ruiz-Páez et al. 2023a). To our knowledge, there have been no studies that have evaluated the effects of noise on hospitalisations in relation with extreme temperature events. In view of the high impact exerted by noise pollution on health, this association must be studied to ensure that prevention strategies can be tailored to the impacts of cold and heatwaves.

### Limitations and strengths

This study has a number of limitations which must be considered. It is important to stress that by virtue of its being an ecological study, conclusions cannot be inferred at an individual level on the basis of the results obtained, owing to the ecological fallacy (Piantadosi et al. 1988).

The pollution data were collected and compiled on the basis of a limited number of monitoring stations, which implies that real exposure at an individual level is not represented, and that what was instead obtained are average values of daily concentrations. This methodological choice was made to address the possibility of population mobility and the dispersion of pollution. With respect to meteorological variables, the data were obtained from a single observatory which, it must be said, is the designated reference point for the Madrid Region.

The standard methodology in these types of studies was used to carry out this study (Samet et al. 2000). To minimise the biases deriving from this methodology, we controlled for different variables, such as the trend of the time series, seasonality and autoregressive nature of the series. It should be noted that no adjustments were made for socio-economic level, which could modify the effect of the associations.

Additionally, the use of hospital admission data could have entailed coding errors. Even so, the foreseeable impact of such possible errors is minimised by having used data drawn from an official source (Instituto Nacional de Estadística, INE) for the entire study period.

This study has a number of strengths, such as the high number of hospital admissions analysed and the variety of specific causes of admission. To our knowledge, this is one of the most up-to-date studies conducted in Spain to examine the impact of air pollution on short-term emergency hospital admissions, taking hot and cold seasons into account. In addition, we controlled for many atmospheric variables and possible confounding factors, with the aim of minimising any possible biases.

Moreover, this study included an analysis of noise pollution, a factor that is little studied despite its high health impact.

Lastly, the results of this study are of great interest for the drawing-up of cold and heatwave prevention plans, by furnishing data that will enable prevention strategies to be tailored to environmental impacts on population health.

### Conclusion

Air pollution, both chemical and noise-related, has a significant impact on health, generating effects similar to those caused by extreme temperatures. This impact is intensified during the summer and winter months, particularly in the case of noise. Hence, it is essential to take chemical and noise pollution levels into account when triggering cold and heatwave alerts. Given the variability in population adaptation to extreme temperature conditions and demographic disparity, small-scale studies are required to adapt prevention plans to the specific needs of the population.

## Electronic supplementary material

Below is the link to the electronic supplementary material.

Supplementary Material 1 (DOCX 1.05 MB)

## Data Availability

The data used for study purposes are confidential.

## Declarations

### Disclaimer

The views expressed by the authors are not necessarily those of the institutions with which they are affiliated.
