# Supplementary material for: Short-term effects of chemical and noise pollution during heat and cold waves on emergency hospital admissions in Madrid
Source: Int J Biometeorol. 2025 Jun 30;69(9):2271–83. doi: 10.1007/s00484-025-02963-y (PMC12479677; doi:10.1007/s00484-025-02963-y)
Supplement: Supplementary file 1 — Supplementary Material 1 (DOCX 1.05 MB) [file 484_2025_2963_MOESM1_ESM.docx]

**Appendix 1**. Map of geographical situation of meteorological, noise and pollution variables quantification. AENA is the public Spanish airport management authority.


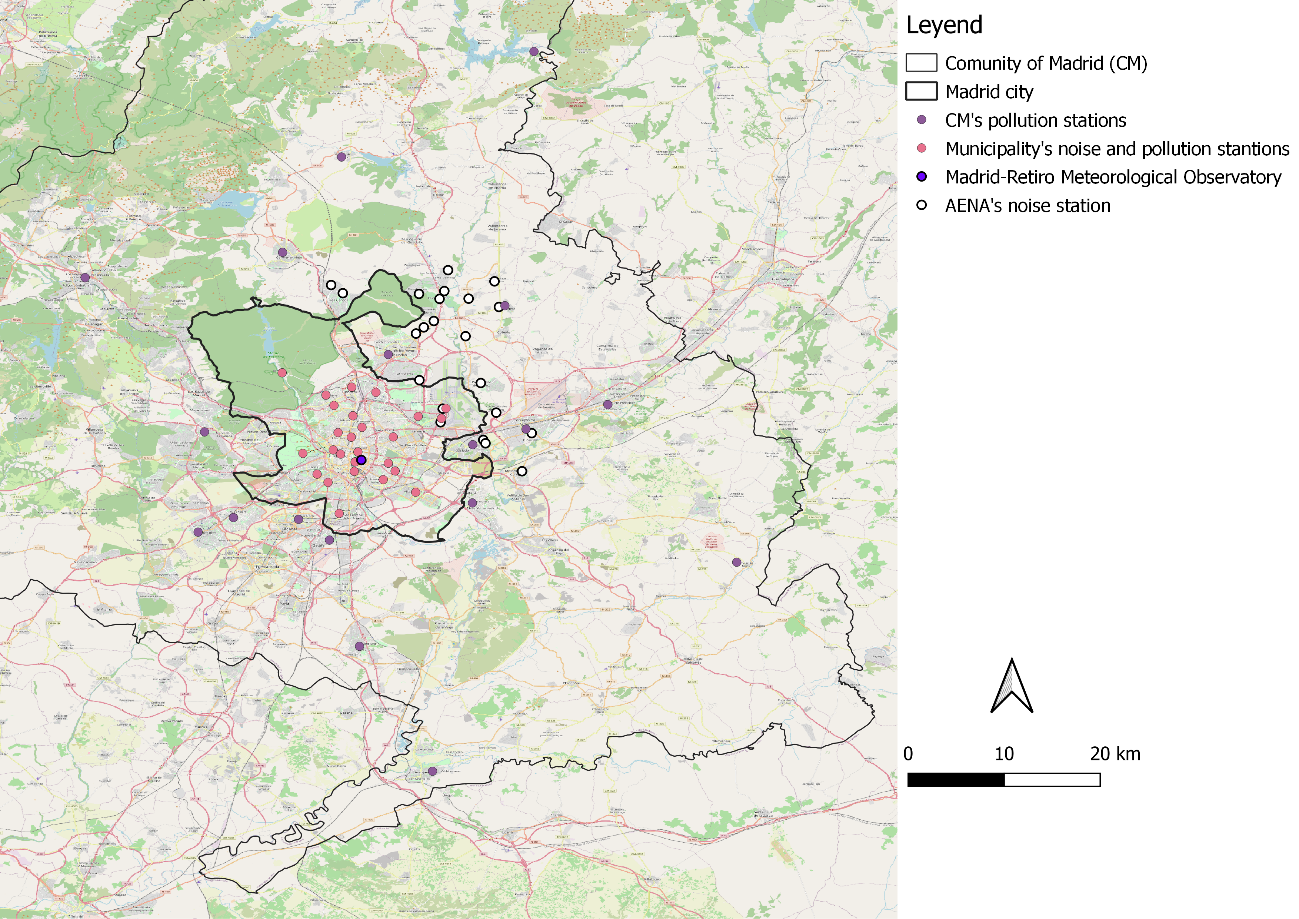


| **Appendix 2**. Effect of environmental variables on emergency hospital admissions | | |
| --- | --- | --- |
|  | RR (95% CI) | AR (95% CI) |
|  |  |  |
| **All respiratory-causes** | |  |
| NO_2_ (3) | 1.00 (1.00 - 1.01) | 0.47 (0.19 - 0.76) |
| O_3_ (0) | 1.23 (1.02 - 1.48) | 18.60 (1.70 - 32.60) |
| O_3_ (1) | 1.29 (1.07 - 1.55) | 22.61 (6.97 - 35.61) |
| Leqd (0) | 1.00 (1.00 - 1.01) | 0.34 (0.17 - 0.51) |
| T.heat (2) | 1.02 (1.00 - 1.03) | 1.52 (0.35 - 2.68) |
| T.cold (3) | 1.02 (1.01 - 1.03) | 1.86 (1.24 - 2.48) |
| T.cold (5) | 1.01 (1.01 - 1.02) | 1.39 (0.78 - 2.00) |
| T.cold (12) | 1.02 (1.01 - 1.03) | 2.02 (1.42 - 2.62) |
| **URTIs** |  |  |
| NO_2_ (4) | 1.02 (1.01 - 1.04) | 2.24 (0.66 - 3.80) |
| Leqn (4) | 1.01 (1.00 - 1.02) | 1.25 (0.27 - 2.22) |
| **Asthma** |  |  |
| NO_2_ (4) | 1.01 (1.00 - 1.03) | 1.38 (0.16 - 2.60) |
| O_3_ (7) | 4.10 (1.77 - 9.51) | 75.61 (43.41 - 89.49) |
| Leqd (4) | 1.01 (1.00 - 1.02) | 0.83 (0.09 - 1.56) |
| T.cold (5) | 1.03 (1.01 - 1.06) | 3.32 (0.66 - 5.92) |
| **Pneumonia** |  |  |
| T.cold (5) | 1.04 (1.01 - 1.07) | 3.43 (0.50 - 6.28) |
| T.cold (11) | 1.04 (1.01 - 1.07) | 3.83 (0.92 - 6.66) |
| **All circulatory causes** | |  |
| O_3_ (4) | 1.34 (1.11 - 1.63) | 25.63 (9.77 - 38.70) |
| O_3_ (8) | 1.23 (1.03 - 1.47) | 18.86 (3.25 - 31.94) |
| Leqd (0) | 1.00 (1.00 - 1.00) | 0.29 (0.10 - 0.48) |
| T.cold (4) | 1.01 (1.00 - 1.02) | 1.05 (0.20 - 1.90) |
| T.cold (6) | 1.01 (1.00 - 1.02) | 1.16 (0.31 - 2.00) |
| T.cold (12) | 1.01 (1.00 - 1.02) | 0.93 (0.14 - 1.71) |
| **Ischaemic heart disease** |  |  |
| T.cold (2) | 1.16 (1.01 - 1.33) | 13.86 (1.15 - 24.93) |
| **MI** |  |  |
| O_3_ (1) | 1.83 (1.08 - 3.12) | 45.46 (7.12 - 67.97) |
| O_3_ (8) | 2.06 (1.21 - 3.49) | 51.37 (17.41 - 71.37) |
| Leqd (0) | 1.01 (1.00 - 1.01) | 0.67 (0.06 - 1.28) |
| T.cold (7) | 1.03 (1.01 - 1.06) | 3.18 (0.63 - 5.66) |
| **ACVA** |  |  |
| O_3_ (1) | 1.76 (1.18 - 2.63) | 43.28 (15.29 - 62.02) |
| Leqd (0) | 1.01 (1.00 - 1.01) | 0.82 (0.34 - 1.29) |
| **Parkinson’s disease** |  |  |
| Leqd (3) | 1.05 (1.01 - 1.10) | 4.89 (0.83 - 8.79) |
| T.heat (1) | 1.20 (1.01 - 1.43) | 16.93 (1.04 - 30.26) |
| **Dementia** |  |  |
| Leqd (0) | 1.03 (1.00 - 1.07) | 3.20 (0.15 - 6.15) |
| **Alzheimer’s disease** |  |  |
| PM_10_ (0) | 1.07 (1.00 - 1.13) | 6.18 (0.37 - 11.64) |
| T.heat (3) | 1.22 (1.07 - 1.38) | 17.74 (6.36 - 27.73) |
| **Multiple sclerosis** |  |  |
| O_3_ (2) | 10.49 (1.48 - 74.33) | 90.47 (32.43 - 98.65) |
| T.cold (6) | 1.16 (1.04 - 1.30) | 13.84 (3.75 - 22.87) |
|  |  |  |
| Relative risks (RRs) and attributable risks (ARs) with their respective 95% Cls for the significant independent variables. Increases for every 10 μg/m3 above the 8-h ozone threshold of 107.5 μg/m3. Lags shown in brackets. Leqd: LAeq,7-23h, Leqn: LAeq,23-7h, Leq24: LAeq,24h. | | |
